# Time series analysis of non-invasive hemodynamic monitoring data in neonates with hypoxic-ischemic encephalopathy

**DOI:** 10.3389/fped.2023.1112959

**Published:** 2023-03-23

**Authors:** Vera Balog, Barbara Vatai, Kata Kovacs, Attila J. Szabo, Miklos Szabo, Agnes Jermendy

**Affiliations:** ^1^ Division of Neonatology, Department of Pediatrics, Semmelweis University, Budapest, Hungary; ^2^ ELKH-SE Pediatric and Nephrology Research Group, Budapest, Hungary

**Keywords:** hemodynamic monitoring, neonates, asphyxia, electrical velocimetry (EV), bioimpedance, hypoxic ischaemic encehalopathy

## Abstract

**Background and aims:**

Hemodynamic instability is common in neonates with hypoxic-ischemic encephalopathy (HIE) undergoing therapeutic hypothermia (TH). Rewarming is a critical period and non-invasive circulatory monitoring may help guide cardiovascular supportive therapy. The aim of the study was to provide a comprehensive analysis of cardiac function parameters during TH and its relation to neurodevelopmental outcome.

**Methods:**

In a prospective, observational study, 26 neonates with moderate-severe HIE were enrolled, born between 2016 and 2019. A hemodynamic monitor based on electrical velocimetry (ICON, Osypka Medical GmbH, Berlin, Germany) was used. Heart rate (HR), stroke volume (SV), cardiac output (CO) data were recorded continuously throughout TH and rewarming. Neurological outcome was assessed at 2 years of age using the Bayley Scales of Infant Development II. edition. Favorable outcome was defined as >70 points on both the psychomotor and mental scales. Time-series analysis was used and features of cardiac function were described to perform logistic regression modeling for outcome prediction.

**Results:**

Fourteen (54%) patients had favorable and 12 (46%) had adverse outcome. Data collection started from median [IQR] of 11.8 [7.0; 24.3] hours (h) of life and lasted until 84.0. [81.8; 87.0] h. During TH, the mean HR of the favorable outcome group was significantly lower than that of the adverse outcome group (86 ± 13/min vs. 104 ± 18/min, *p* = 0.01). During rewarming HR increased similarly in both groups. SV was unaffected by rewarming, and showed a slowly increasing trend. SV of the favorable outcome group was significantly higher compared to the adverse outcome group (1.55 ± 0.23 ml/kg vs. 1.29 ± 0.30 ml/kg, *p* = 0.035). In line with this, CO was similar in both groups (136 ± 27 ml/kg/min vs. 134 ± 36 ml/kg/min), and a significant 25% increase in CO was observed during rewarming. Based on multiple regression modeling, HR during TH was independently associated with neurological outcome (*p* = 0.023).

**Conclusion:**

Based on continuous hemodynamic monitoring, patients with adverse outcome have lower SV and higher HR to achieve similar CO to patients with favorable outcome during TH. HR during hypothermia is independently associated with the neurodevelopmental outcome.

## Introduction

Perinatal asphyxia and the subsequent hypoxic-ischemic encephalopathy (HIE) is one of the leading causes of mortality and permanent neurological deficit among term neonates (1). Total body therapeutic hypothermia (TH) has been proven to reduce mortality and severe neurodevelopmental disability in children with moderate to severe HIE (2–4). In addition to the neurovascular impairment, these infants often present with multiorgan failure, hemodynamic instability and systemic hypotension. The underlying pathophysiology may be variable among patients: hypovolemia, peripheral vasoconstriction during TH and vasoregulatory impairment, as well as decreased cardiac output (CO) due to myocardial ischemia could all contribute to hemodynamic instability, and may lead to suboptimal cerebral perfusion (5–7). It has been suggested that to achieve the best possible neurodevelopmental outcome, optimization of hemodynamic management is necessary (8).

The rewarming period after TH requires special attention, since the hemodynamic status changes rapidly to meet the increased metabolic demand of the body. Several studies have examined the changes during rewarming. Previously, it was described that lower heart rate (HR) during TH is associated with favorable outcomes (9, 10), and CO significantly increases during rewarming, due to an increase in HR (11). Investigating cardiac function could help in choosing the optimal vasoactive support, and ideally, it requires continuous assessment of HR, stroke volume (SV) and CO. Since targeted neonatal echocardiography is still not widely available, and provides only intermittent analysis, non-invasive hemodynamic monitors may be a reasonable alternative. These monitors are routinely used in the adult population and have been successfully tested in children and neonates (12–14).

Electrical velocimetry (EV) is the most widely used method of non-invasive hemodynamic monitoring. The technique is based on the changes in the impedance of the red blood cells during the cardiac cycle, using four electrodes attached to the patient’s skin. A meta-analysis of 24 studies found that EV cannot replace echocardiography for measuring the absolute values of CO, however, it can complement monitoring in the intensive care units and is suitable for following trends and aiding clinical decision-making (14). Importantly, it has been used in neonates undergoing TH, and reflected the expected hemodynamic changes (13).

Despite the successful application of non-invasive hemodynamic monitors in HIE patients, previous studies have not analysed the time-series features of the continuously registered HR, SV and CO data. Analysis of single, cross-sectional segments of data will not reflect fluctuations of generalized trends and may lead to information loss for individual patients. Furthermore, detailed time-series analysis of cardiac function may reveal associations between acute disease severity and long-term neurodevelopmental outcome. Thus, the primary aim of this study was to provide a comprehensive time-series analysis of HR, SV and CO during TH and the subsequent rewarming period and to identify the potential differences in the monitored cardiac function values in groups with distinct neurodevelopmental outcomes.

## Materials and methods

### Study design and patient population

**Figure 1:**
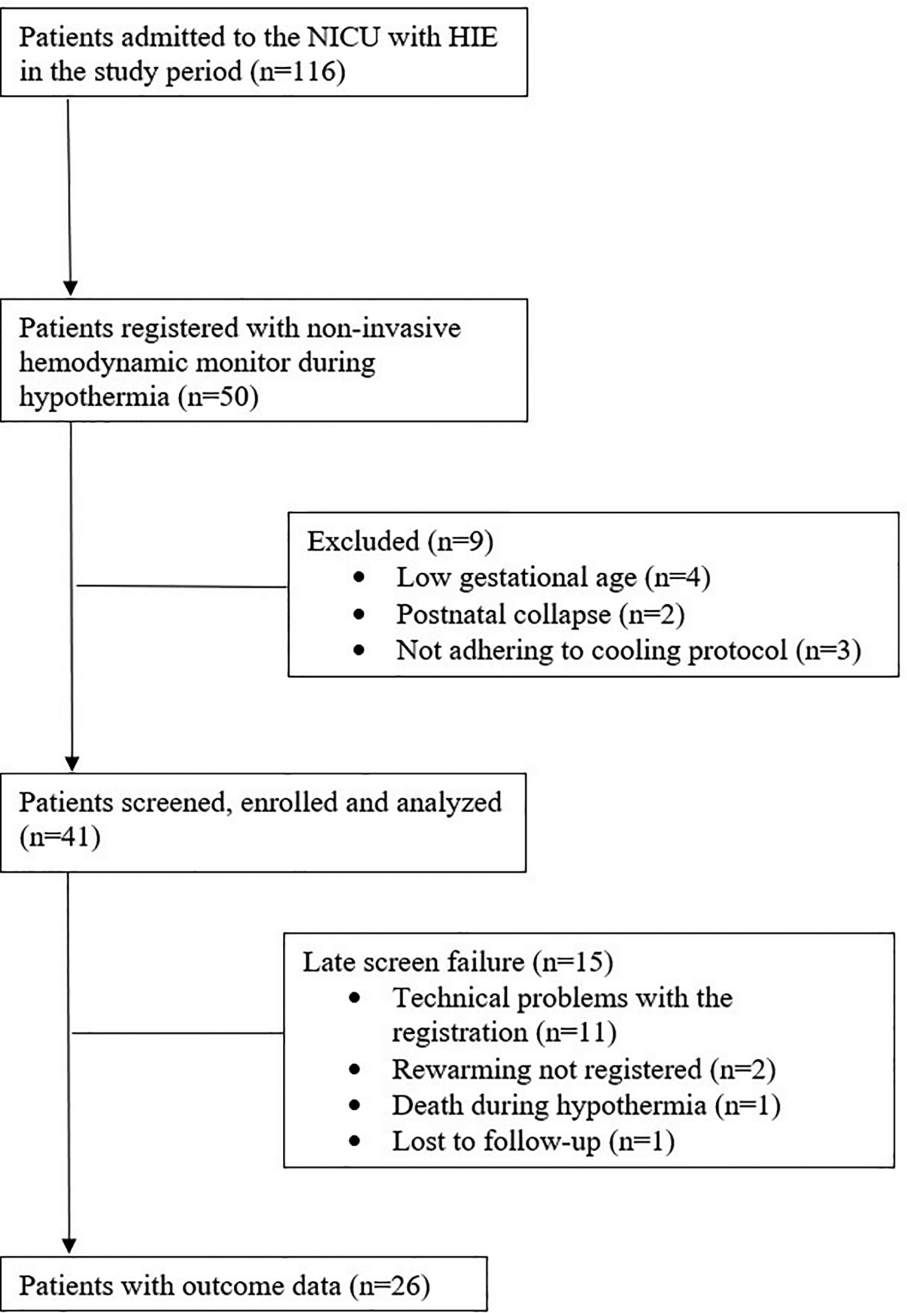
Flowchart showing the enrollment procedure of the study.

This was a prospective, observational study conducted in the Neonatal Intensive Care Unit (NICU) of the Department of Pediatrics, Semmelweis University Budapest, Hungary. The study was approved by the Ethics Committee of the National Medical Research Council (ETT-TUKEB 11790-2/2016/EKU and 7469-12/2021/EUIG). We enrolled patients who were born between July 2016 and February 2019, were diagnosed with moderate-to-severe HIE as described by Azzopardi et al. (4), and were cared for in our level III NICU, a regional cooling center. All patients were outborn. Inclusion criteria were (1) whole body TH for moderate-to-severe HIE, (2) non-invasive hemodynamic monitoring during TH. Exclusion criteria were (1) gestational age of <37 week, (2) postpartum asphyxia, (3) cooling initiated >6 h of life or other cooling protocol violations, (4) technical problems with data registration (equipment interference or sensor problems), (5) congenital malformations, (6) death during TH, (7) missing neurodevelopmental follow-up examination. Patient recruitment and exclusions are shown on Figure 1. Eight patients with persistent pulmonary hypertension (PPHN) receiving high-frequency ventillation (HFO) were excluded for technical problems with data registration, because of the interference between the non-invasive hemodynamic monitor and the HFO ventilation mode. None of the patients had sepsis in our cohort.

### Equipment

Non-invasive hemodynamic monitoring was applied using the ICON electrical velocimetry device (Ospyka Medical GmbH, Berlin, Germany). The ICON continuously recorded HR, SV and CO and measurements were averaged in every 60 s leading to a time-series dataset with minute frequency. For data analysis, CO and SV values were divided by the infant’s birth weight. Measurements are shown in the following format: ml/kg/min for CO, ml/kg for SV, and beats/minutes for HR.

The quality of the measured signal was verified using the signal quality index (SQI) values of the monitor. Low-quality data points of the measurement signal (SQI <80) were discarded from the statistical analyses, as recommended previously (15).

### Clinical care

Whole-body hypothermia treatment was induced as described in the TOBY trial; initiated within the first 6 h of life, maintained for 72-hours, followed by gradual rewarming (4, 16). All patients were mechanically ventilated during the hypothermia treatment according to our institutional clinical protocol. Respiratory management of individual cases is decided by the attending neonatologist, but our institutional guideline is to use SIMV-VG (synchronised intermittent mandatory ventilation with volume guarantee) mode without pressure support, with a PEEP (positive end-expiratory pressure) of 5 cmH_2_O. The target expired tidal volume is 4–5 ml/kg initially, which is adjusted later based on blood gases.

All patients were sedated with morphine sulfate infusion (loading dose of 100 µg/kg, followed by continuous infusion of 10 µg/kg/hour). Cerebral activity was recorded using continuous single-channel (biparietal, P3-P4) amplitude integrated electroencephalography (aEEG) monitoring (Olympic CFM 6,000 monitor; Natus Medical or EEG-1200 K; Nihon Kohden). Recording was started as soon as possible but always before 6 h of age and continued for the duration of therapeutic hypothermia and rewarming. The aEEG background activity was scored according to the Hellstrom–Westas (17) criteria, and aEEG normalization was defined as attaining continuous normal voltage activity.

Cardiovascular management was based on the mean arterial pressure targeted to reach the value of the gestational age in weeks (18). If hypotension occurred, vasopressor agents were initiated based on the decision of the clinician. Dopamine infusion was started usually as first-line therapy at a rate of 6 µg/kg/min, and if necessary, the dose was increased in 2 µg/kg/min increments up to 20 µg/kg/min. As per local protocol, 0.5 mg/kg hydrocortisone every 6 h was used frequently as a second-line therapy, in cases of suspected relative adrenal insufficiency (based on clinical and laboratory findings) or vasopressor-resistant hypotension (4, 16, 19). If needed, further vasopressor-inotropes were added including dobutamine, epinephrine, norepinephrine or milrinone. Vasotropic-inotropic score [VIS] was calculated as: dopamine dose (µg/kg/min) + dobutamine dose (µg/kg/min) + 100 × epinephrine dose (µg/kg/min) + 10 × milrinone dose (µg/kg/min) + 10,000 × vasopressin dose (unit/kg/min) + 100 × norepinephrine dose (µg/kg/min), it was calculated in every hour during hypothermia and rewarming, which allowed for direct comparison of inotropic needs in each patient over time (20). Clinicians were blinded to the ICON measurements.

MRI was performed at the Medical Imaging Center, Semmelweis University, Budapest, using a 3 Tesla Philips Achieva scanner (Philips Medical System, Best, The Netherlands). T1-, T2-, and diffusion-weighted MRI with apparent diffusion coefficient measurements were performed. MRI scans were classified using the scoring system developed by Weeke et al. (21).

### Neurodevelopmental outcome

Bayley Scales of Infant Development (II. Edition) (22) was used at two years of age to evaluate the neurodevelopmental outcome undertaken by a trained psychologist blinded to the clinical history. Favorable outcome was defined by scoring ≥70 points on both the mental development index (MDI) and psychomotor development index (PDI) scales, while adverse outcome was defined by scoring <70 points on either scale or death. Patients who were unable to complete the Bayley exam due to severe neurological impairment were automatically assigned to the adverse outcome group.

### Statistical analysis

Descriptive statistics were expressed as mean ± standard deviation (SD) or median with interquartile ranges [IQR] for continuous variables and as number with percentage (%) for categorical variables. Fisher’s exact test and Wilcoxon rank sum test were used to compare groups on categorical and continuous variables, respectively.

Time-series features were derived from ICON signals as follows: (1) A common time window was selected with relation to the start of rewarming to ensure that the same period is analyzed for every patient; (2) individual HR, SV, and CO signals in the selected time window were plotted on the y axis against time on the x axis by applying LOWESS (locally weighted scatterplot smoothing) both for each patient separately and at the group-level; (3) signals with visible breakpoints at the start of rewarming were modeled by segmented robust linear regression (as implemented by the lmrob function of the robustbase R package (23), that is, two separate slope coefficients were estimated per signal (one for hypothermia and one for rewarming period) besides the intercept (representing the estimated value at the onset of rewarming); (4) signals with no sign of rewarming-triggered change were modeled by robust linear regression as a function of time, resulting in two features describing the signal: intercept at the start of rewarming period and slope.

To control for the vasoactive-inotrope need during the study period, a simple linear regression model was built to model VIS change over time in the selected time window, and the estimated VIS score at the start of rewarming was used afterwards. Due to skewed data, VIS scores were logarithmically transformed before calculations.

A multiple logistic regression model was developed to ascertain the effects of the extracted ICON time-series features on neurodevelopmental outcome. Since the CO signal is a function of HR and SV, only SV, HR, and VIS features were included in the model.

Finally, receiver operating curve statistics were calculated with the area under the curve analysis to evaluate the predictive power of the logistic regression model.

We accepted *p* < 0.05 as a level of significance. R Statistical Software, Version 4.2.1 (R Foundation for Statistical Computing, Vienna, Austria) was used for data analysis and plotting.

## Results

### Population

Born between July 2016 and February 2019, a total of 116 HIE patients received TH in our institute, of whom 50 patients were monitored with ICON. For the present analysis, we excluded patients who had low gestational age (4 patients) or had postnatal collapse (2 patients), or were not adhering to cooling protocol (3 patients). Another 15 patients were excluded due to late screen failure: technical problems with data registration (11 patients); rewarming was not registered (2 patients); death during hypothermia (1 patient); and lost to follow-up (1 patient). After these exclusions, 26 patients were included in the study, of whom 14 (54%) patients had favorable and 12 (46%) had adverse neurodevelopmental outcome at two years of age (performed at a median 19 [18–20] months). Flow diagram of study enrollment is shown in Figure 1. Demographics and clinical characteristics of the patients are shown in Table 1. Patients in the adverse outcome group had more severe brain injury on the MRI (performed at a median 4 [4; 5] days of life), and aEEG normalization occurred less frequently, and later than in patients with favorable outcome.

**Table 1:** Baseline characteristics of the study population.

	Favorable outcome (*n* = 14)	Adverse outcome (*n* = 12)
Gestational age (week)	39.5 [38.0–40.0]	39.0 [37.8–40.0]
Male sex (%)	7 (50%)	5 (42%)
Birth weight (g)	3,360 [3,000–3,610]	3,180 [2,960–3,610]
**First blood gas analysis**
pH	7.00 [6.82–7.14]	6.94 [6.72–7.12]
pCO2 (mmHg)	56.0 [33.4–84.9]	40.1 [32.9–80.0]
BE (mmol/L)	−16.0 [−19.0–(−13.5)]	−21.4 [−24.3–(−16.7)]
Lactate (mmol/L)	6.0 [3.7–6.7]	8.1 [4.9–11.0]
Apgar 1′	3 [1–4]	2 [1–2]
Apgar 5′	5 [2–7]	4 [2–5]
Apgar 10′	7 [5–8]	6 [3–7]
Bayley II scores MDI	86 [76–112]	50 [50–50]
Bayley II scores PDI	98 [90–104]	50 [50–91]
**MRI studies^a^**
Total score of brain MRI	2 [0–14.5]	25 [15–37]
**Presence of injuries on MRI**
Deep gray matter injury	5 (36%)	11 (91%)
White matter/cortex injury	8 (57%)	10 (83%)
Cerebellar injury	3 (21%)	5 (42%)
**aEEG registration^b^**
Normalization of aEEG <48 hs	11 (79%)	3 (25%)
Time of aEEG normalization (hours of life) (*n* = 14)	18 [5–24]	35 [33–37]

Data are shown as median [IQR] or numbers (%).

pCO2, partial pressure of carbon dioxide; BE, base excess; MRI, magnetic resonance imaging; aEEG, amplitude integrated electroencephalogram; MDI, Mental Developmental Index; PDI, Psychomotor Developmental Index.

Bayley Infant Developmental Scale (II. edition) was used at 18–23 month of age to evaluate the outcome. Patients who were unable to complete the Bayley exam due to severe neurological impairment were automatically assigned to the adverse outcome group.

^a^ Brain MRI scans were available in 24 patients, and were scored according to the scoring system published by Weeke et al.

^b^ aEEG recordings were available in 23 patients, and normalization was defined as attaining continuous normal voltage background activity, as described by Hellstrom-Westas et al.

### Hemodynamic characteristics

**Figure 2:**
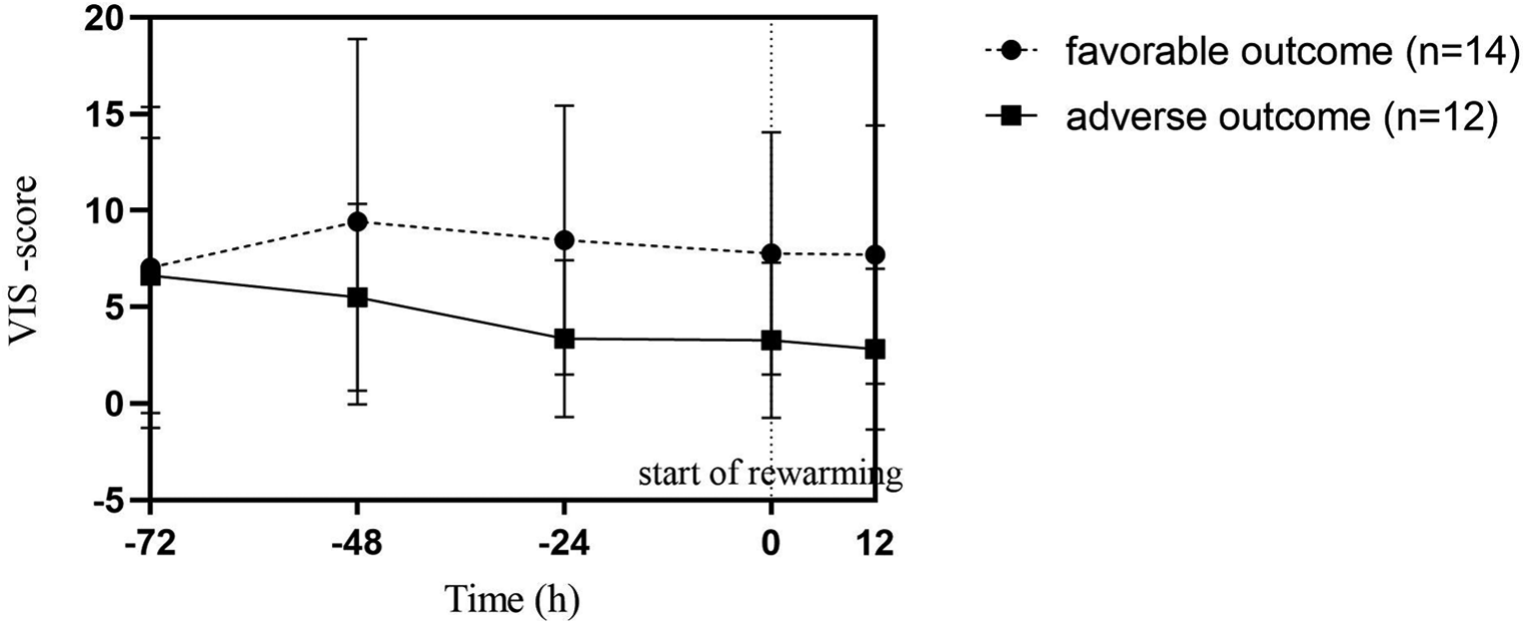
Vasoactive-inotropic (VIS) scores during therapeutic hypothermia in favorable and adverse neurodevelopmental outcome groups. Point 0 of the X axis marks the start of rewarming. Datapoints represent the median of individual patient scores with interquartile ranges. Each patient score was calculated by averaging hourly scores of the previous period. There was no statistical difference between the two outcome groups.

To treat hypotension, vasotropic-inotropic agents were administered in 23 (88%) patients. Dopamine was used in almost all patients, only 2 patients received a combination of vasoactive therapy and 1 patient received norepinephrine along with dopamine and dobutamine. There was no difference between the need for vasoactive drugs between the two groups, as shown in Table 2. We used a continuous VIS-score (19) to assess possible differences in vasoactive treatment between the groups that may influence cardiac function. The hourly VIS-score during TH was median 5.4 [3.5; 11.0] in the favorable outcome group and 5.3 [0.0; 7.3] in the adverse outcome group (*p* = 0.049), as shown in Figure 2. In addition, 50% of patients received hydrocortisone therapy as well.

**Table 2:** Vasoactive drugs administered in the study population.

Vasopressor-inotropes administered	Favorable outcome (*n* = 14)	Adverse outcome (*n* = 12)	*p-*value
None	1 (7%)	2 (17%)	0.58
Dopamine alone	11 (79%)	8 (67%)	0.67
Dobutamine alone	0	1 (8%)	0.46
Combined therapy: Dopamine + Dobutamine	1 (7%)	1 (8%)	1.00
Combined therapy: Dopamine + Dobutamine + Norepinephrine	1 (7%)	0	1.00
Hydrocortisone	6 (43%)	7 (58%)	0.70
Hourly VIS scores	5.3 [0.0; 7.3]	5.4 [3.5; 11.0]	0.049

Data are shown as numbers (%) or median [IQR].

VIS, vasoactive-inotrope score.

### Preprocessing of ICON measurements

Descriptive statistics of the raw ICON signals are shown in Table 3. Recordings started at a median 11.8 [7.3; 24.0] hours of life and ended at 84.0 [82.0; 86.8] hours. The median length of ICON measurements and number of valid data points, with signal quality index higher than 80, were similar in the adverse and favorable outcome groups.

**Table 3:** Technical details of ICON measurements.

	All patients (*n* = 26)	Favorable outcome (*n* = 14)	Adverse outcome (*n* = 12)	*p*-value
Data points per patients	1,686 [1,489; 1,733]	1,710 [1,332; 1,724]	1,640 [1,556; 1,759]	0.70
Ratio of SQI < 80	0.04 [0.02; 0.10]	0.07 [0.02; 0.11]	0.03 [0.02; 0.05]	0.30
Length of total measurement (hours)	74 [59; 79]	75 [67; 79]	70 [58; 79]	0.70
Length of hypothermia phase (hours)	61 [51; 67]	63 [58; 67]	58 [49; 65]	0.40
Length of rewarming phase (hours)	10.6 [8.7; 13.6]	10.1 [8.6; 12.2]	11.6 [9.2; 14.5]	0.40

Data are shown as median [IQR].

SQI, signal quality index.

**Figure 3:**
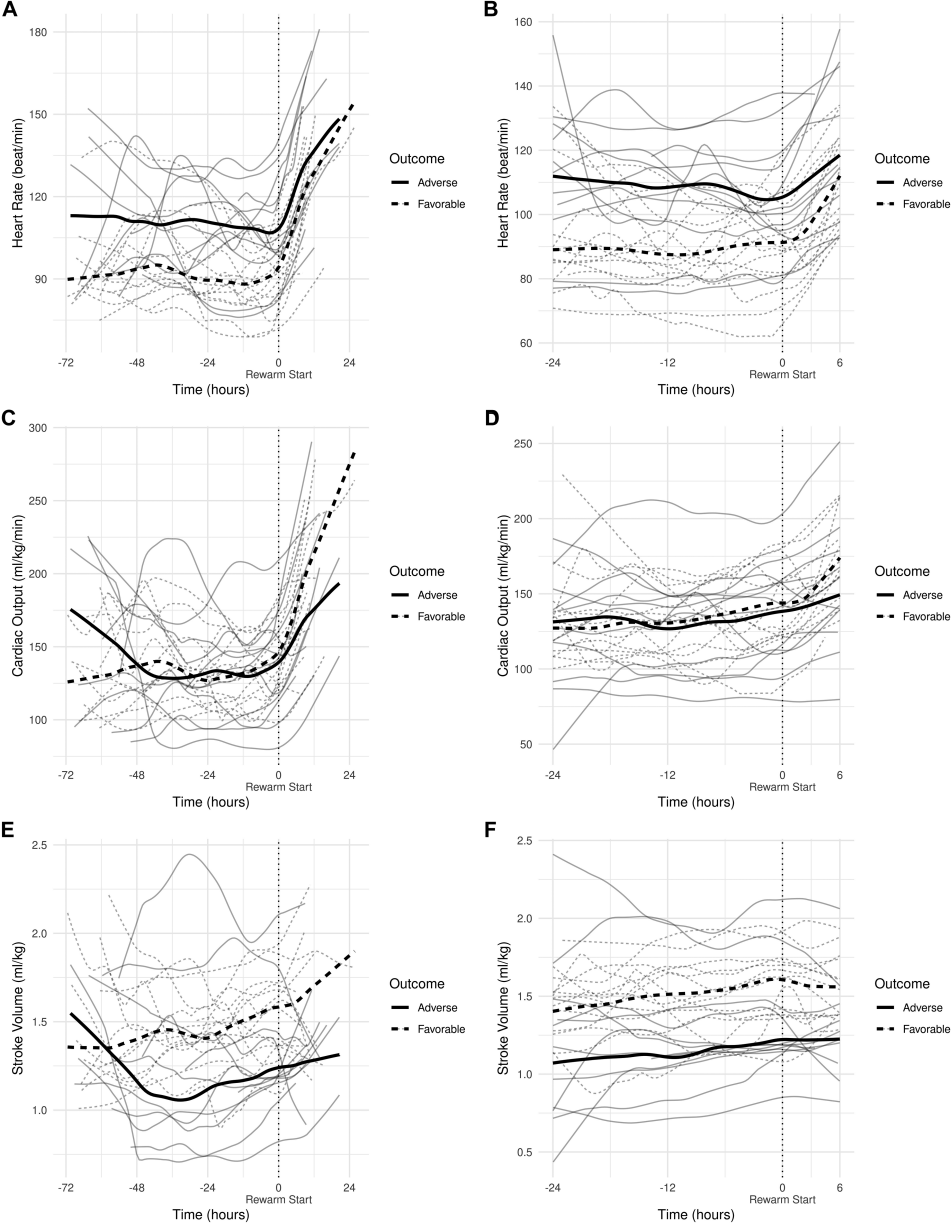
(**A**) smoothed curves of the heart rate time-series variable. For smoothing, LOWESS (locally-weighted scatterplot smoothing) was applied on every individual separately (thin lines), or on each group separately (thick lines). (**B**) smoothed curves of heart rate of the individual patients in different groups cut at the −24 h +6 h of therapeutic hypothermia. (**C**) Smoothed curves of the cardiac output time-series variable. (**D**) smoothed curves of cardiac output of the individual patients in different groups cut at the −24 h +6 h of therapeutic hypothermia. (**E**) Smoothed curves of the stroke volume time-series variable. (**F**) smoothed curves of stroke volume of the individual patients in different groups cut at the −24 h +6 h of therapeutic hypothermia.

All individual recordings were aligned to the onset of rewarming. The lengths of pre-rewarming and rewarming phases did not differ between groups (Table 3), but there was substantial variation at the individual level. Therefore, a common cutoff was applied to ensure that time-series features were extracted from the same time windows in relation to rewarming. Cutoff points were determined by visual inspection of the signals smoothed individually by locally weighted scatterplot smoothing (see Figure 3). Because the signals were relatively stable in the −24 h to +6 h interval, and all but two patients had full coverage in that interval (one having shorter pre-rewarming, the other shorter rewarming phases), individual recordings were cut at −24 and +6 h with relation to the onset of rewarming.

In line with our expectations, based on the results of previous studies (10, 11, 13), the slopes of HR and CO signals clearly changed after the start of rewarming (see Figure 3), whereas no apparent breakpoint could be detected for SV signals. Thus, segmented robust linear regression was performed for each patient to extract HR and CO features, and non-segmented robust linear regression models were fitted to SV signals (resulting in 3 features [2 slopes and 1 intercept] per patient for HR and CO, and 2 features [1 slope, 1 intercept] for SV signals, respectively). Thus, feature extraction yielded a total of 8 features per patient for describing the time-series HR, CO, and SV data, their descriptive statistics are presented in Table 4. We also present the comparison of estimated values for the cardiac function parameters at −24 h (hypothermia), 0 h (start of rewarming), and at 6 h (end of rewarming) (Table 5).

**Table 4:** Estimated individual time-series features.

Characteristics	Favorable outcome (*n* = 14)	Adverse outcome (*n* = 12)	Difference	*p-*value
**HR at hypothermia (beat/min)**	**86** (**13)**	**104** (**18)**	**17**	**0**.**01**
HR slope at hypothermia (beat/min/h)	−0.17 (0.56)	−0.27 (0.52)	−0.10	0.60
HR slope at rewarming (beat/min/h)	4.03 (2.30)	3.03 (2.20)	−1.0	0.30
CO at hypothermia (ml/kg/min)	136 (27)	134 (36)	−2.3	0.90
CO slope at hypothermia (ml/kg/min/h)	0.33 (0.98)	0.12 (0.63)	−0.21	0.50
CO slope at rewarming (ml/kg/min/h)	5.13 (3.08)	3.70 (3.29)	−1.4	0.30
**SV at hypothermia (ml/kg)**	**1.55** (**0.23)**	**1.29** (**0.33)**	−**0**.**26**	**0**.**035**
SV slope (ml/kg/h)	4 (8)	2 (7)	−2.2	0.50
log_VIS-score	1.44 (1.40)	0.79 (1.34)	−0.65	0.20

Data are shown as mean (standard deviation).

Statistically significant *p*-values are highlighted in bold.

HR, heart rate; CO, cardiac output; SV, stroke volume; VIS, vasoactive-inotrope score.

**Table 5:** Comparison of estimated values of cardiac function at −24 h, 0 h, and 6 h.

Characteristic	Favorable outcome (*n* = 14)	Adverse outcome (*n* = 12)	Difference	*p*-value
**HR (beat/min)**
24 h before the onset of rewarming	91 (15)	110 (18)	20	0.007
At the onset of rewarming	86 (13)	104 (18)	17	0.01
6 h after the onset of rewarming	111 (19)	122 (24)	11	0.20
**CO (ml/kg/min)**
24 h before the onset of rewarming	128 (22)	131 (34)	2.8	0.80
At the onset of rewarming	136 (27)	134 (36)	−2.3	0.90
6 h after the onset of rewarming	167 (27)	156 (44)	−11	0.50
**SV (ml/kg)**
24 h before the onset of rewarming	1.45 (0.23)	1.25 (0.46)	−0.21	0.20
At the onset of rewarming	1.55 (0.23)	1.29 (0.33)	−0.26	0.035
6 h after the onset of rewarming	1.57 (0.25)	1.30 (0.31)	−0.27	0.023

Data are shown as mean (standard deviation).

HR, heart rate; CO, cardiac output; SV, stroke volume.

### HR, CO, and SV features

**Figure 4:**
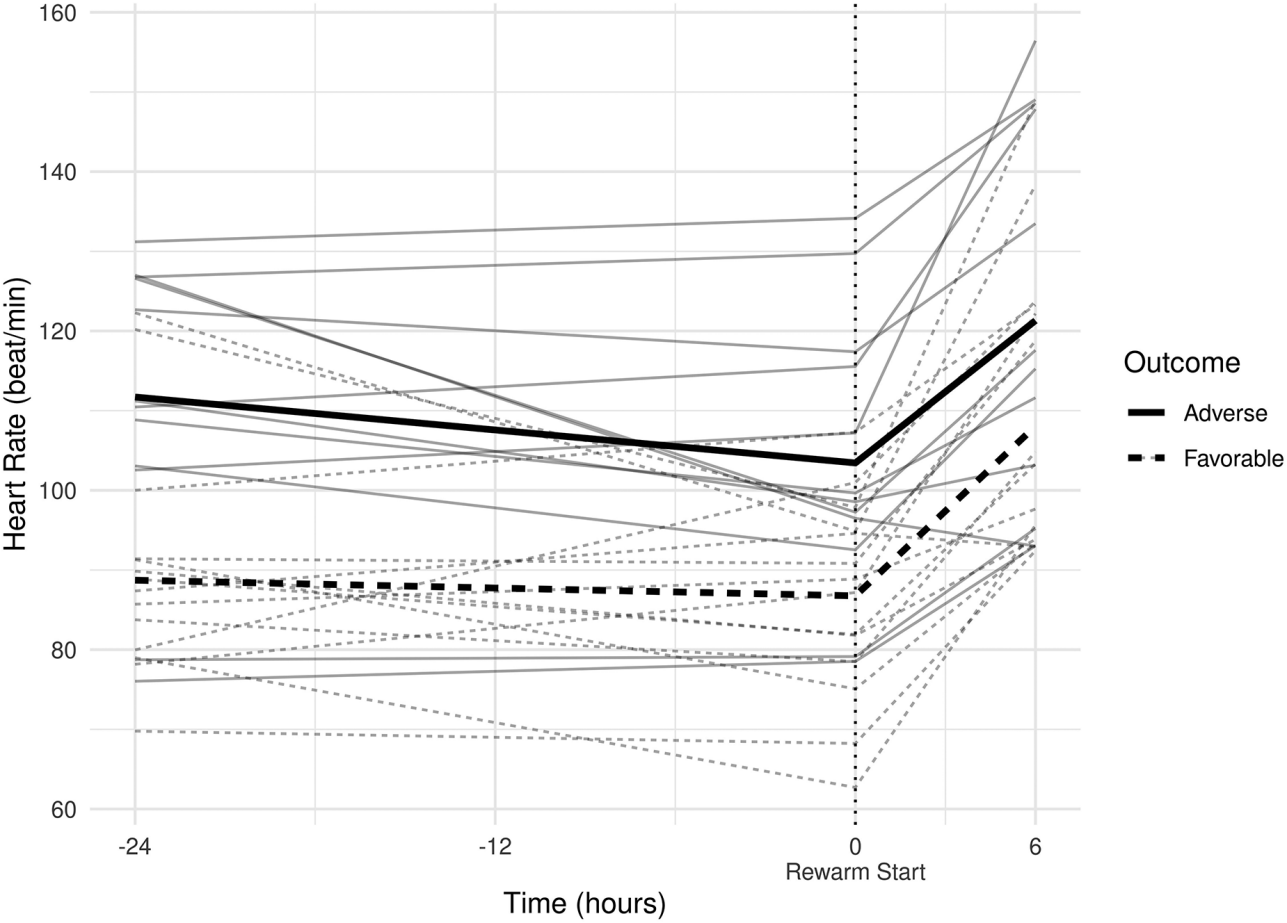
Estimated individual heart rate regression lines and group-level averages of graphs.

**Figure 5:**
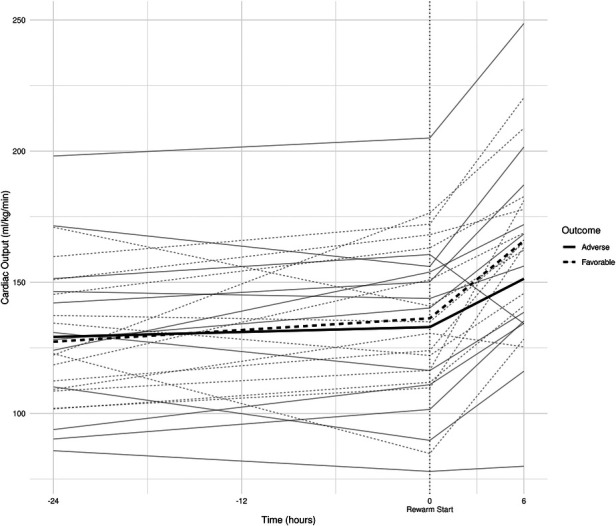
Estimated individual cardiac output regression lines and group-level averages of graphs.

**Figure 6:**
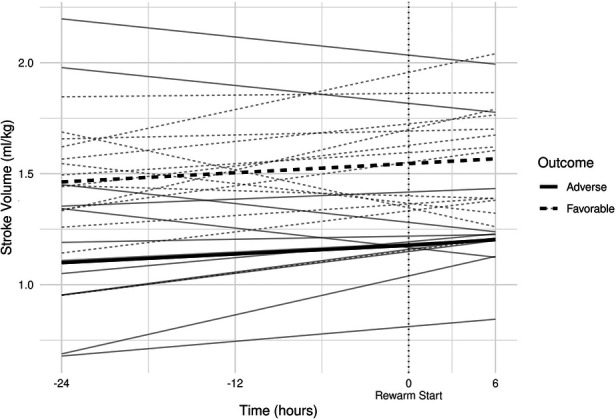
Estimated individual stroke volume regression lines and group-level averages of graphs.

The estimated individual time-series features (that is, the individual regression lines) and their group-level averages are depicted in Figures 4–6. In general, the estimated *slopes* in the two groups were highly similar: HR and CO values were relatively stable in hypothermia, and started to increase during rewarming, with an estimated 25% increase in CO. In contrast, SV values showed only a slight increase in the analyzed time window. Regarding the estimated *intercepts*, that is, the estimated values of the signals at the onset of rewarming, the HR was significantly higher in the adverse group compared to the favorable outcome group (104 vs. 83 beat/min), and SV was lower (1.29 vs. 1.55 ml/kg, respectively).

### Prediction

**Figure 7:**
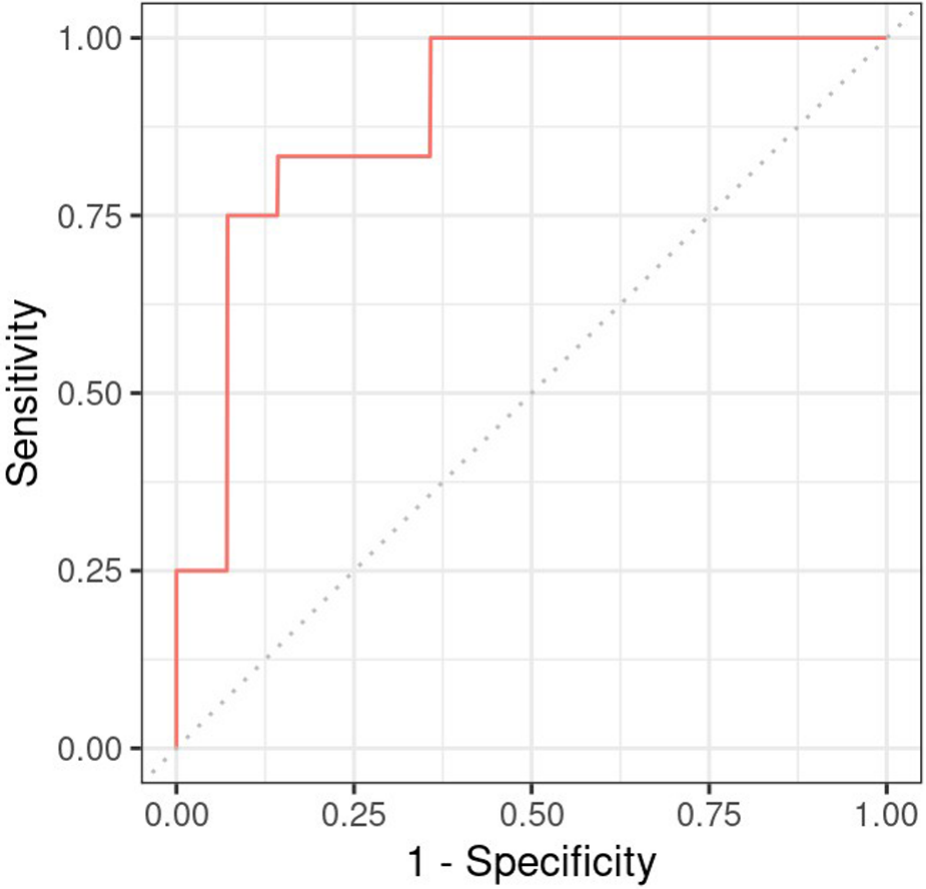
Receiver operating charateristics curve of the regression model constructed for neurodevelopmental outcome with heart rate during hypothermia as predictor.

Next, a logistic regression model was developed to ascertain the effects of the cardiac function features on neurodevelopmental outcome. As predictors, we included the three HR features (slope in hypothermia, value at the onset of rewarming, and slope during rewarming), two SV features (slope in the −24 to +6 h period and value at the onset of rewarming), and the VIS score (logarithm at the onset of rewarming). The CO was not included, as it is a function of HR an SV. The multiple regression yielded that only HR value at the onset of rewarming had a significant effect on the outcome, with higher heart rate increasing the odds of adverse outcome (odds ratio: 3.2, CI: 1.38–10.81, *p* = 0.020). The result suggests that a 10 beat/minute difference in HR at the onset of rewarming is associated with an increase of 3.2 in the odds of developing adverse neurological outcome (see Table 6). By testing all models which consist of all possible combinations of the predictor variables, we confirmed it is only the HR intercept which remains significant (data not shown). The AUC of the model is 0.893 (Figure 7).

**Table 6:** Logistic regression using the features of the cardiac function graphs.

Feature	OR	95% CI	*p-*value
**Baseline HR (10 beat/min)**	**3**.**20**	**1.38, 10.81**	**0**.**023**
Slope of HR during TH	0.05	0.003, 1.21	0.129
Slope of HR during rewarming	0.71	0.38, 1.17	0.207
Baseline SV	1.64	0.01, 405.17	0.843
Slope of SV	0.86	0.68, 1.02	0.132
Log VIS-score	0.67	0.21, 1.61	0.407

Multiple logistic regression for prediction of neurodevelopmental outcome (reference category: favorable outcome), based on cardiac function features and VIS score as predictors.

Statistically significant *p-*value is highlighted in bold.

HR, heart rate; SV, stroke volume; TH, therapeutic hypothermia; VIS-score, vasotropic-inotropic score; OR, odds ratio; CI, confidence interval.

## Discussion

This was a prospective study, assessing cardiac functions continuously with electrical velocimetry in asphyxiated infants treated with TH. Time-series data analysis revealed that patients with favorable neurodevelopmental outcome have significantly lower HR and higher SV achieving similar CO compared to patients with adverse outcome. A multiple regression model controlling for the administered vasoactive drugs suggested that HR during hypothermia can be an independent predictor of the long-term outcome, emphasizing the association between encephalopathy and cardiovascular haemodynamics.

Physiological sinus bradycardia has been observed during therapeutic hypothermia in asphyxiated infants, which is considered acceptable for the reduced metabolic rate, with 60% decreased cardiac output (5). During rewarming, however, the augmentation of cardiac output is expected, with increasing systolic blood pressure and a concurrent decrease in systemic vascular resistance (24). In line with these physiological observations, we have seen two phases in the slopes of HR and CO signals, with a clear breakpoint at the start of rewarming. Importantly, no apparent breakpoint could be detected for the SV signals, suggesting that CO increase during rewarming is driven by HR, not by SV, a finding consistent with a recent study (11).

Previous studies have described that the severity of HIE correlates with multi-organ failure (25, 26). It was also reported that the hypoxic insult causes myocardial damage, right ventricular dysfunction, and altered transitional circulation (27, 28). However, much less is known about the association between cardiac dysfunction and long-term outcomes of HIE patients. A recent study, using similar non-invasive hemodynamic monitoring, showed that infants with brain injury on MRI had lower CO driven by a lower SV rather than HR; although none of the differences reached statistical significance (13). In the present study, we detected similarly lower SV trends. These findings suggest that a more severe hypoxic insult causes both significant brain injury and profound myocardial injury, which presents with a decreased SV. In addition, we also noted higher HR in patients with adverse outcome, leading to a CO that was similar in patients with favorable outcome. Several small and large, registry-based studies described that higher heart rate during cooling was associated with adverse outcomes (10, 29, 30). A recent publication also reported on the use of continuous hemodynamic monitoring of HIE neonates (31). The authors described that while HR increased gradually, SV remained stable during TH, but increased during rewarming in patients with moderate HIE. As CO is a function of HR and SV, we suggest that higher HR in patients with more severe hypoxic insult is likely a compensatory mechanism to maintain adequate CO in face of decreased SV. Another publication also proposed that the appropriate HR to provide sufficient CO may depend on myocardial contractility and preload (32). However, it is also plausible that higher HR reflects autonomic dysfunction in patients with severe hypoxic insult (30).

Assessment of cardiovascular dysfunction is warranted in all HIE patients treated with TH as hemodynamic instability is common, and may contribute to ongoing injury (33). Cerebral perfusion is related directly to CO in the absence of major shunts and there is a preferential distribution of CO towards the brain, especially in severe HIE patients. In addition, vasoparalysis, caused by the hypoxic insult, renders the brain susceptible to fluctuations in blood flow. In previous studies, it was described that hypotension was associated with an increased risk of severe brain injury (34) and decreased right ventricular performance was linked to adverse neurodevelopmental outcome in these newborns (27). On the other hand, iatrogenic hypertension due to cardiovascular medications may exacerbate reperfusion injury in the brain. Continuous hemodynamic monitoring, such as electrical velocimetry, is a valuable tool, because it is non-invasive and gives continuous information on cardiovascular function trends. Importantly, electrical velocimetry was shown to be comparable with echocardiography in estimating CO (35). Our study confirms the feasibility of ICON monitoring during TH, and its application may be particularly useful when titrating cardiovascular medications, especially during the rewarming period to avoid fluctuations in CO and cerebral blood flow (36).

An important novelty of our study is that we used high-frequency, time-series data analysis instead of the conventional approach of evaluating physiological variables at distinct timepoints. The advantage of feature extraction (ie. creation of additional variables or “features” from raw data) from enormous quantities of patient-specific time-series data is that we could harness information about the variable’s development over time without losing information, leading to more accurate predictions on outcome. In addition, based on these features, mathematical algorithms may be developed in the future, in larger patient cohorts, that can warn of imminent clinical deterioration (37).

Naturally, there are limitations of this study. First, relatively low number of patients were included in the study, however, we believe that the high-frequency data recording compensates for the within-subject variability to some extent. Second, ICON recordings were not started at a uniform time-point in all patients, but we managed to align the measurements by selecting a “0” time point at the start of rewarming. Third, we showed that HR during cooling is an independent predictor of outcome, however, several other factors should be considered beside myocardial ischemia that may influence the actual HR. These factors include chronotropic drugs, pain, or sedation. Our unit use inotropes more frequently than others (38) and it may affect HR and SV. Hence, we attempted to control for the effect of cardiovascular medications by including the VIS score in the regression analysis. Of note, many patients also received hydrocortisone therapy, and it may increase catecholamine sensitivity (8). Fourth, we did not measure troponin levels in our patients, which could have been an objective biochemical marker of myocardial injury. Finally, we did not perform a systematic evaluation with echocardiography in these patients, however, the accuracy of the ICON device was studied previously (13, 35) and true precision was described to be 31.6% (35). Due to the lack of normative data or intervention thresholds for non-invasive CO measurements, we suggest the ICON device to be used only for following trends of cardiovascular hemodynamics (39).

## Conclusion

According to our data, HIE patients with adverse neurodevelopmental outcome have higher HR and lower SV achieving similar CO values as the ones with favorable outcome. We suggest that HR during hypothermia is independently associated with neurodevelopmental outcome at 2 years of age in patients with HIE.

## Data Availability

The raw data supporting the conclusions of this article will be made available by the authors, without undue reservation.

## Author's note

Short oral presentation at EAPS Congress 2022, Barcelona. Title: Continuous non-invasive hemodynamic monitoring of neonates undergoing hypothermia treatment.
